# Evaluation of the levels of pain and discomfort of piezocision-assisted flapless corticotomy when treating severely crowded lower anterior teeth: a single-center, randomized controlled clinical trial

**DOI:** 10.1186/s12903-019-0758-9

**Published:** 2019-04-16

**Authors:** Omar Gibreal, Mohammad Y. Hajeer, Bassel Brad

**Affiliations:** 1 Oral and Maxillofacial Surgery Department, University of Damascus Dental School, Damascus, Syria; 2 Department of Orthodontics, University of Damascus Dental School, Damascus, Syria; 3 Department of Oral and Maxillofacial Surgery, University of Damascus Dental School, Damascus, Syria

**Keywords:** Piezoelectric, Visual analog scale, Flapless piezocision, Patient-centered outcomes, Severe crowding alignment

## Abstract

**Background:**

No randomized controlled trial (RCT) has compared flapless piezocision-assisted corticotomy in the extraction-based orthodontic decrowding of lower anterior teeth with the conventional treatment in terms of pain, discomfort and acceptability. Therefore, the aim of this trial was to compare piezocision-based orthodontic decrowding of lower anterior teeth following premolar-extraction with the conventional orthodontic treatment regarding levels of pain, discomfort, and patients’ satisfaction.

**Methods:**

A parallel-group RCT was conducted on 34 patients with severely crowded lower anterior teeth. Subjects were randomly allocated to either the experimental (ExpG) or the control group. Piezoelectric corticotomies were performed on the labial surfaces of the alveolar bone in the anterior region in the ExpG. Levels of pain, discomfort, swelling, difficulties of mastication, swallowing and jaws movement limitation were recorded on a Visual Analog Scale (VAS) at 1, 7, 14 and 28 days. In the ExpG, patients were also asked to rate their level of satisfaction following acceleration. Two-sample t tests were employed to detect significant differences.

**Results:**

No statistically significant differences were found between the two groups at one day following treatment commencement regarding pain, discomfort, difficulties of mastication, swallowing and limitation in jaws movement (*P* = 0.082, 0.367, 0.062, 0.446, 0.359; respectively). However, a statistically significant difference was found between the two groups regarding the perception of swelling at the first-day assessment (*P* = 0.011). No statistically significant differences were detected between the two groups at 7 days regarding the five previously mentioned variables. There was a drop down to zero level at two weeks and four weeks following treatment onset for all variables. The level of satisfaction in the ExpG had a mean value of 86.47 (±22.47) and all patients were positive towards recommending the surgical intervention to a friend.

**Conclusions:**

No significant differences in the levels of pain and discomfort were found between the ExpG and the control group for all variables except for the perception of swelling at one day following intervention. Patient-centered outcomes revealed a high level of acceptance and satisfaction with this technique.

**Trial registration:**

This trial was registered at Clinical Trials.gov (Identifier NCT02975765).

**Electronic supplementary material:**

The online version of this article (10.1186/s12903-019-0758-9) contains supplementary material, which is available to authorized users.

## Background

Many surveys have declared that 70–95% of patients complain from pain caused by orthodontic appliances [1]. Orthodontic pain has been described as a main reason for patients’ withdrawal and cessation of treatment [2–4]. Furthermore, some authors have reported that pain and discomfort have been more intense in adults and could be one of the main reasons beyond discouraging them from undergoing orthodontic treatment [5–7].

Dental crowding is considered one of the most common types of malocclusion [8]. Methods of conventional treatments vary between extraction and non-extraction approaches [9]. Non-extraction therapy is usually used to resolve mild to moderate conditions, while extraction method is usually used to aid in the correction of moderate to severe cases. Extraction-based treatment could last for a long period of time and has it been documented that it would take up to 35 months [10].

Reducing orthodontic treatment time is one of the main goals for orthodontists and patients especially adults [11]. Prolonged orthodontic treatment times have possible complications such as root resorption, periodontal disease, caries in addition to the undesirable pain accompanying the treatment procedures at different stages [12]. One survey described pain as the greatest dislike during treatment and fourth among major pretreatment fears and concerns [13].

Several techniques have been proposed to accelerate orthodontic treatment and the most common approaches were the surgical ones [14] such as: interseptal alveolar surgery [15], osteotomy [16], corticotomy [17, 18], dentoalveolar distraction [19, 20], periodontal distraction [21], *corticision* [22, 23] and Piezocision technique [24–26]. The acceptance of traditional corticotomy-assisted orthodontics among patients was generally low, mainly because of the invasive procedures and postoperative discomfort and complications [27]. Flapless piezocision-assisted corticotomy has been found to have various advantages over the traditional methods of corticotomy and is considered a promising minimally invasive tooth acceleration technique [28].

Although various techniques of piezocision flapless corticotomy have been reported to be successful in practice [25, 29], scientific evidence on their accompanying pain, discomfort, acceptance and quality of life is little in the literature and more high-quality RCTs investigating those aspects are required [28, 30]. Three trials evaluated the levels of pain associated with minimally invasive surgical procedures [29, 31, 32]. Alikhani et al. assessed pain and discomfort levels during canine retraction after applying micro-osteoperforations, while Mehr and Charavet studied pain levels during the acceleration of piezocision-assisted non-extraction tooth decrowding cases. Therefore, it seems to be that decrowding strategies based on extraction plans accompanied with acceleration modalities have not been evaluated yet in terms of pain, discomfort and acceptability.

The present randomized controlled clinical trial aimed to compare piezocision-assisted orthodontic decrowding of lower anterior teeth following premolar-extraction with the conventional orthodontic treatment regarding levels of pain and discomfort as well as patients’ post-treatment satisfaction.

## Methods

### Study design

This study was a two-arm, parallel group randomized controlled trial comparing the levels of pain and discomfort between piezocision-assisted orthodontic treatment and the traditional method of aligning crowded lower anterior teeth. Participants were recruited from the Departments of Orthodontics at Damascus University Dental School between March 2016 and February 2017. The Local Research Ethics Committee Approval was obtained (UDDS-2455-15,032,015/SRC-4991). This trial was registered at Clinical Trials.gov (Identifier NCT02975765) and was funded by the University of Damascus Dental School Postgraduate Research Budget (Ref no: 83054206785DEN).

### Sample size estimation

Sample size was calculated using the G*power 3.1.7 software with an alpha level of 0.05, a power of 80%. The smallest difference requiring detection in pain level was assumed to be 25 mm on a visual analog scale (VAS) with a standard deviation of 23.75 mm (from a previous study [31]); therefore, a sample size of 32 patients was required for both groups (i.e., *n* = 16 for each group).

### Patient selection, recruitment, and follow-up

**Fig. 1:**
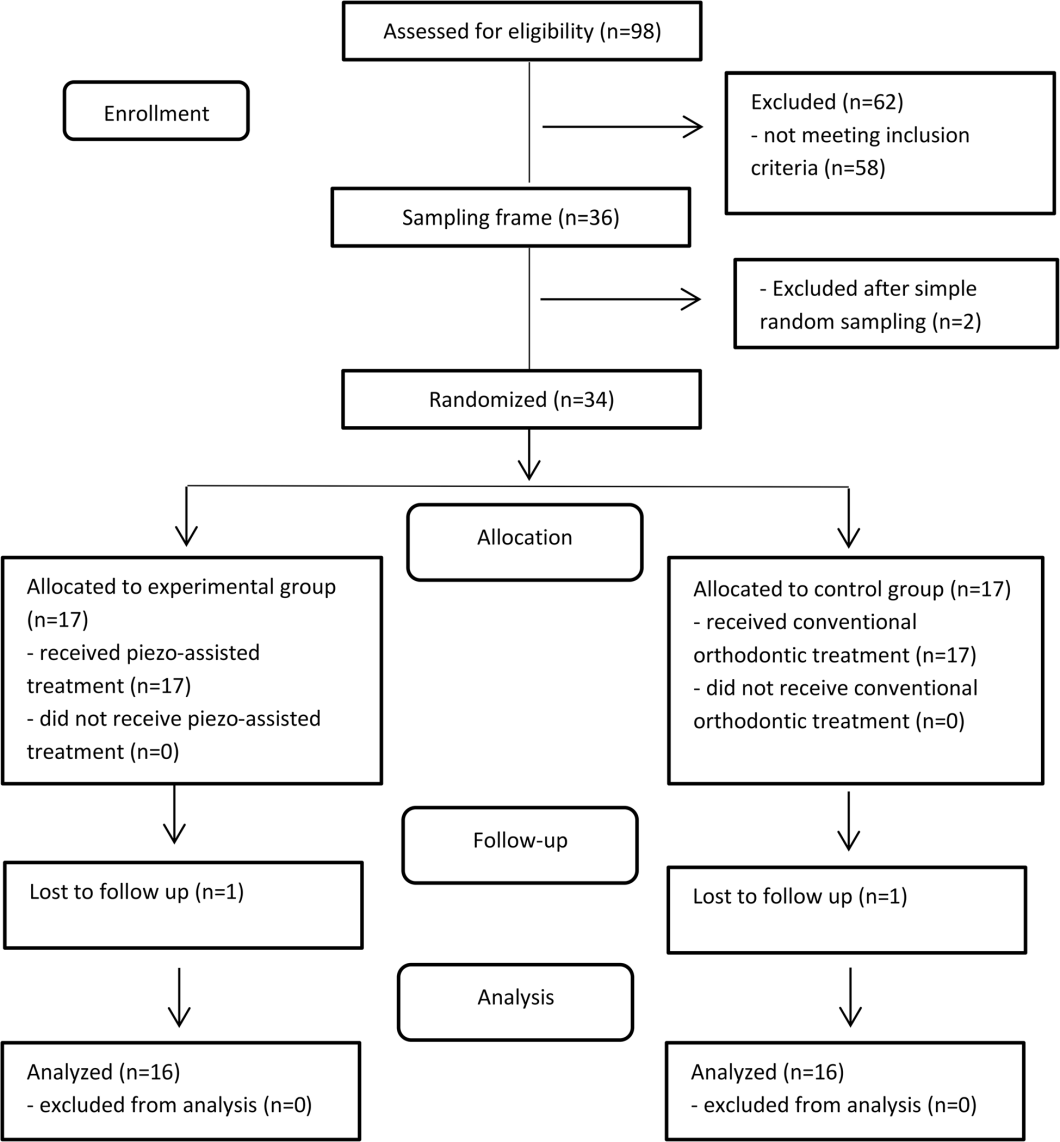
CONSROT 2010 flow diagram of patients’ recruitment and follow-up

Patients were selected from the Department of Orthodontics at University of Damascus Dental School. The treatment plan of 98 severe dental crowding patients was reviewed, but the number of patients who met the inclusion criteria and agreed to participate in this study after the acquaintance with the information sheet was 40. According to a priori sample size calculation which indicated the need for 32 patients. Thirty-four subjects were equally and randomly assigned to the two groups; this number was chosen for any potential drop-out after the commencement of the trial. Patients’ selection and follow-up is shown in Fig. 1.

Information sheets were distributed to all patients and informed consents were obtained. The inclusion criteria were: (1) Adult healthy patients from both sexes within an age range 17–24 years; (2) absence of previous orthodontic treatment; (3) Class II division I patients requiring first upper premolars extraction; (4) completion permanent dentition (except of third molars); (5) absence of medications intake that interfere with pain perception for at least one week before the beginning of the treatment;(6) good oral hygiene and healthy periodontium which was evaluated clinically (probing depth ≤ 3 mm, no radiograph evidence of bone loss, plaque and gingival index ≤1 according to Silness and Loe [33]).

Exclusion criteria were: (1) Medical conditions that affect tooth movement (Corticosteroid, NSAIDs, Bisphosphonates, Hyperparathyroidism, Osteoporosis and Uncontrolled diabetes); (2) medical, social and psycho contraindications to oral surgery; (3) presence of primary teeth in the mandibular arch; (4) missing permanent mandibular teeth (except third molars); (5) patients had previous orthodontic treatments; (6) poor oral hygiene or concurrent periodontal disease: probing depth ≥ 4 mm, radiographic evidence of bone loss, gingival index > 1, plaque index > 1 [33].

### Randomization and allocation concealment

Patients were assigned to the experimental group or the control group with an allocation ratio of 1:1 using a software-generated list of random numbers. Allocation sequence was concealed using sequentially numbered, opaque, sealed envelopes which were opened only after the completion of premolars extraction. First group received piezocision-assisted orthodontic treatment, whereas the second group received conventional orthodontic treatment (Fig. 1). The generation of random allocation sequence, participants’ enrollment and assignment to the two groups were performed by one of the academic staff not involved in this research.

### Orthodontic procedures

All subjects underwent conventional orthodontic treatment with fixed appliances. One week following first-premolar extraction, fixed orthodontic appliances with an MBT prescription and 0.022-in. slot height (Master Series®, American Orthodontics™, Sheboygan, WI USA) were bonded. For both groups, the archwire sequence used was 0.014-in. NiTi followed by 0.016-in., 0.016 X0.022-in. and 0.017 X 0.025-in. NiTi, and finally 0.019 X 0.025-in. stainless steel [25]. Change in archwires was performed when it was felt that an improvement had occurred in teeth positions and there was a possibility of inserting the next archwire without exerting excessive force on the engaged teeth. Treatment was considered finished when LII was less than 1 mm, indicating complete alignment of the teeth and the feasibility of inserting the final archwire passively into all brackets [34].

### Piezocision surgical procedure

**Fig. 2:**
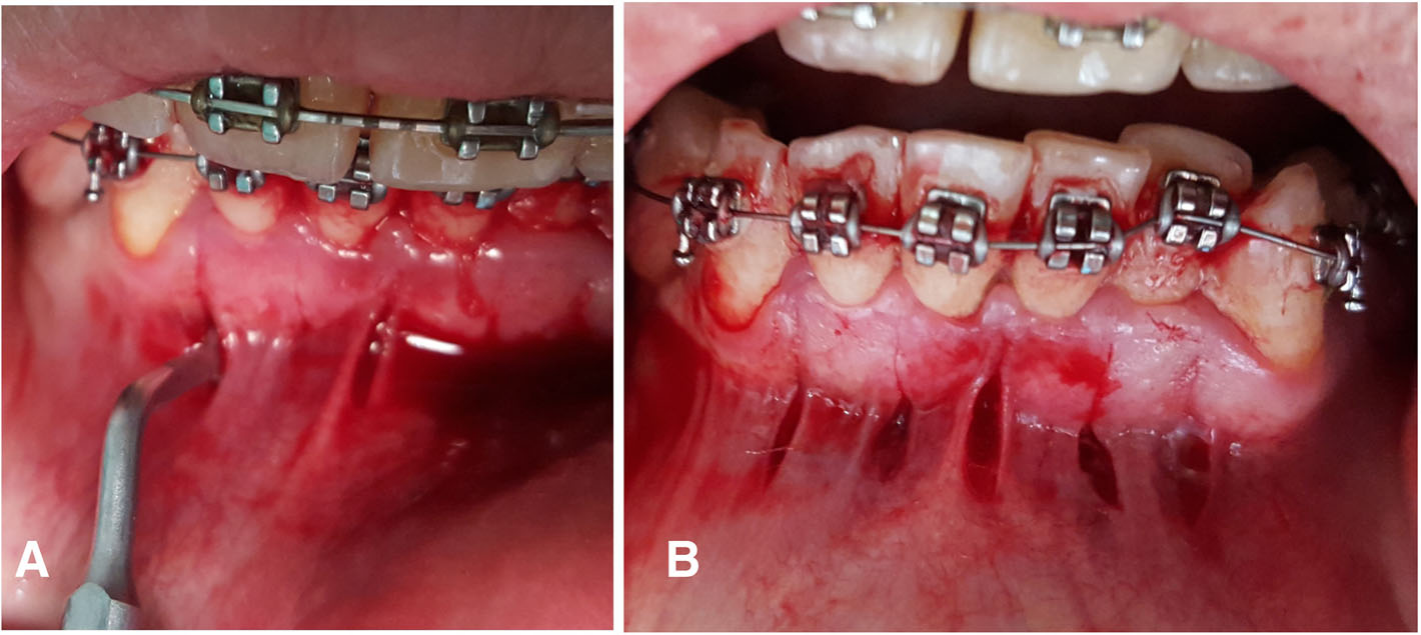
The minimally invasive piezocision intervention. **a**: The instrument used for performing the cortical cuts. **b**: Five vertical corticotomies were performed in the lower anterior segment

Radiographic metal guides were placed on the archwire for the experimental group subjects as a guide to make precise mucoperiosteal incisions avoiding periodontal ligaments and teeth roots. Patients were asked to rinse with Chlorhexidine Gluconate 0.12% for 1 min immediately before the surgical intervention, then local Infiltration was injected (lidocaine hydrochloride 2% with epinephrine 1:80,000). The surgical protocol was performed as described by Dibart [35]. The incisions began 4 mm below the papilla to prevent any further gingival recessions, then a PT1 periotome (Hu-Frieday Mfg.Co., Chicago, USA) was used to confirm the incisions lines without elevating the periosteum and raising any flaps. Vertical 5 to 8-mm-long and 3-mm-deep corticotomies were performed utilizing a Piezosurgical Microsaw (Implant Center™ 2, Satelec, France) with a BS1 cutting tip and irrigation solution pump 80 ml/m (Fig. 2). No subsequent sutures were performed and the surgical side was covered by a piece of Iodoform gauze.

After the surgical procedure, patients were prescribed: a soft diet for 3 days after the surgery, rinses with Chlorhexidine Gluconate 0.12% twice a day for 1 week, ice packs for the first 12 h following surgery; and were instructed to take one or two tables of Panadol® (acetaminophen; 500 mg) when they suffer from moderate/severe pain provided that questionnaires are completed first. No anti-inflammatory drugs were prescribed.

Experimental patients check-ups were scheduled a day after the procedure ensuring absence of postoperative complications, and were followed up every two weeks for orthodontic treatment sequence.

### Outcome measures

The outcome measures for both groups included levels of pain, discomfort, and swelling, difficulties of mastication, swallowing and jaws movement limitation. The questionnaire was given to patients at one day, 7 days, 14 days, 28 days following the onset of treatment (Additional file 1). All patients were instructed to rate their levels on a Visual Analog Scale (VAS) questionnaire. A line of 100-mm length was used with the left side representing no pain, discomfort, swelling, difficulties of mastication, swallowing or jaws movement limitation (i.e., score = 0) and the right side representing the worst pain, highest levels of pain, discomfort, swelling, difficulties of mastication, swallowing and jaws movement limitation (i.e., score = 100). Each patient was asked to put a vertical mark on the line at a point which best represented the perceived levels of the aforementioned variables. Patients were instructed not to take any analgesic during pain assessment period. In addition, experimental patients’ acceptance of the received intervention, the treatment duration, and whether they would recommend the procedure to friends were assessed after treatment completion. Patients were also asked to record any consumption of analgesics and number of tables used (Additional file 2).

### Statistical analysis

Parametric tests were used since Anderson-Darling Normality tests showed normal distributions of the collected data. Two-sample t-tests were used to detect significant differences between the two groups at each assessment time. Single blinding was employed in this trial regarding outcome measure assessment and data analysis. All statistical analyses were performed by one of the coauthors (MYH) using Minitab® Version17 (Minitab Inc., Pennsylvania, USA).

## Results

Initially, 34 patients were enrolled in this study. Unfortunately, one patient in each group dropped out before the end of the trial due to personal reasons (i.e. moving to another city), leaving 16 patients in each group for the data analysis stage.

Basic sample characteristics are given in Table 1, while descriptive statistics of the sample regarding the evaluated variables at one day, 7 days are given in Tables 2 and 3, respectively. No descriptive statists are given regarding the variables assessed at 14 days and 28 days since all obtained values dropped down to zero. The results of significance tests regarding pain, discomfort, and swelling, difficulties of mastication, swallowing and jaws movement limitation according to assessed time points are given in Table 4.

**Table 1:** Basic sample characteristics

Group	Gender n (%)	*P*-value^a^	Mean Age (SD)	Min. Age	Max. Age	*P*-value^b^
Control	Male 7 (43.75%)	0.531	21.27 (1.87)	18	24	0.092
	Female 9 (56.25%)					
Experimental	Male 6 (37.5%)		20.86 (1.98)	17	23	
	Female10 (62.5%)					
All sample	32 (100%)		21.03 (1.96)	17	24	

*Min* minimum, *Max* maximum

^a^employing chi-square test

^b^employing two-sample t test

**Table 2:** Descriptive statistics of patients-centered variables at one day after first orthodontic archwire insertion in the two groups using visual analog scales (*n* = 16 for each group)

Variable	Group	Mean	SD	SEM	Min	Q1	Median	Q3	Max
Pain	Exp.	32.06	15.92	3.86	10.00	20.00	30.00	40.00	70.00
	Control	21.76	12.62	3.06	5.00	10.00	20.00	30.00	50.00
Discomfort	Exp.	28.82	18.67	4.53	10.00	10.00	20.00	40.00	70.00
	Control	23.53	14.87	3.61	5.00	10.00	20.00	35.00	50.00
Swelling	Exp.	26.18	12.44	3.02	10.00	15.00	30.00	37.50	50.00
	Control	13.82	12.20	3.44	0.00	2.50	10.00	20.00	40.00
Mastication	Exp.	33.82	22.47	5.45	5.00	20.00	30.00	45.00	70.00
	Control	20.59	13.79	3.35	0.00	10.00	20.00	30.00	50.00
Swallowing	Exp.	4.71	3.10	2.42	0.00	0.00	0.00	20.00	20.00
	Control	4.11	1.72	0.71	0.00	0.00	0.00	10.00	10.00
Limitation	Exp.	22.06	12.88	3.12	5.00	10.00	20.00	35.00	40.00
	Control	17.06	7.21	1.95	0.00	0.00	0.00	15.00	60.00

*Exp*. experimental group, *SD* Standard Deviation, *SEM* Standard Error of the mean, *Min* minimum, *Q1* first quartile, *Q3* third quartile, *Max* maximum

**Table 3:** Descriptive statistics of patients-centered variables at 7 days after first orthodontic archwire insertion in the two groups using visual analog scales (*n* = 16 for each group)

Variable	Group	Mean	SD	SE	Min	Q1	Median	Q3	Max
Pain	Exp.	6.47	7.45	1.81	0.00	0.00	5.00	10.00	30.00
	Control	1.176	2.811	0.682	0.00	0.00	0.00	0.00	10.00
Discomfort	Exp.	6.03	7.80	1.02	0.00	0.00	5.00	10.00	20.00
	Control	2.35	5.04	1.22	0.00	0.00	0.00	5.00	20.00
Swelling	Exp.	1.765	3.509	0.851	0.00	0.00	0.00	2.50	10.00
	Control	0.882	1.965	0.477	0.00	0.00	0.00	0.00	5.00
Mastication	Exp.	2.94	1.87	0.48	0.00	0.00	0.00	10.00	30.00
	Control	1.18	1.17	0.70	0.00	0.00	0.00	20.00	20.00
Swallowing	Exp.	0.00	0.00	0.00	0.00	0.00	0.00	0.00	0.00
	Control	0.00	0.00	0.00	0.00	0.00	0.00	0.00	0.00
Limitation	Exp.	2.353	3.999	0.970	0.00	0.00	0.00	5.00	10.00
	Control	0.882	2.643	0.641	0.00	0.00	0.00	0.00	10.00

*Exp*. experimental group, *SD* Standard Deviation, *SEM* Standard Error of the mean, *Min* minimum, *Q1* first quartile, *Q3* third quartile, *Max* maximum

**Table 4:** The results of significance tests of the observed patients-centered variables on visual analog scales at 1 and 7 days following the onset of treatment (n = 16 in each group)^a^

Variable	One day						One week					
	Mean difference	SD	95% CI for difference		*P*-value	Significance	Mean difference	SD	95% CI for difference		*P*-value	Significance
			Min	Max					Min	Max		
Pain	10.29	14.3	0.26	20.33	0.082	NS	5.29	5.63	1.36	9.23	0.093	NS
Discomfort	5.29	16.8	−6.50	17.09	0.367	NS	4.12	6.35	−0.33	8.56	0.068	NS
Swelling	12.35	13.4	3.03	21.68	0.011	*	0.882	2.84	−1.10	2.869	0.372	NS
Mastication	13.24	18.6	0.21	26.26	0.062	NS	4.71	8.52	−1.25	10.66	0.117	NS
Swallowing	7.06	6.3	2.65	11.47	0.446	NS	–	–	–	–	–	–
Limitation	10.59	10.7	3.08	18.09	0.359	NS	1.47	3.38	−0.90	3.84	0.215	NS

^a^Two sample t test; * significant at *P* < 0.05; NS: Non-significant

No statistically significant differences between the two groups were found at one day following treatment commencement regarding pain, discomfort, difficulties of mastication, swallowing and limitation in jaws movement (*P* = 0.082, 0.367, 0.062, 0.446, 0.359, respectively). However, a statistically significant difference was found between the two groups regarding the perception of swelling (*P* = 0.011). The experimental subjects developed a higher feeling of swelling with a mean of 26.18 (±3.02) compared to the control group ($\overline{x}$ =13.82 ± 3.44). Moreover, no statistically significant differences between the two groups were detected at 7 days regarding pain, discomfort, swelling, difficulties of mastication and jaws movement limitation (*P* = 0.093, 0.068, 0.372, 0.117, 0.215, respectively). Difficulties of swallowing levels reached zero in both groups at 7 days after treatment onset. Experimental subjects’ acceptance mean value was 86.47 (±22.47) after treatment completion, and all of them answered that they would recommend this acceleration procedure to a friend. Surprisingly, none of the patients in either group took pain killers during the leveling and alignment stage.

## Discussion

This is the first RCT having the objective of evaluating the levels of pain and discomfort between flapless piezocision technique and the traditional method in the alignment of severely crowded lower anterior teeth in extraction cases.

The VAS was used as a tool to measure pain perception because of its superiority on other scales and has been used in previous studies [29, 36, 37].

Patient-centered outcomes were first measured at 24 h following treatment commencement in order to avoid the analgesic effect of the local anesthesia in the experimental group. Hence, our results showed that there were no significant differences in pain and discomfort levels between the two groups at one day after treatment commencement (*P* = 0.082; *P* = 0.367; respectively), nor at 7 days (*P* = 0.093, *P* = 0.068; respectively). Then they reached zero value after 14 and 28 days. This could be explained by the precision of the piezosurgery micro-saw that permitted a safe cutting mode with maximum control and the possibility of conducting selective cutting designs preserving root integrity and reducing post-surgical pain that is well known with conventional cutting tools (i.e. surgical burs) [38–40] . Similarly, two previous trials reported no significant differences in pain and discomfort levels after applying piezocision flapless corticotomies in terms of orthodontic decrowding acceleration compared to conventional orthodontic treatments [29, 31]. Both aforementioned trials adopted visual analog scales to evaluate patient-centered outcomes. However, both studies included non-extraction cases compared to extraction cases in the current trial. When evaluating post-intervention pain at the same assessment time among the current trial and the aforementioned studies (i.e. at 7 days postoperatively), the experimental subjects’ levels of pain was greater in the Charavet’s and Mehr’s RCTs than those of the current trial (i.e., mean values: 60 mm, 30.28 mm, and 6.47 mm respectively).

In this study, mean pain levels in the experimental group was 32.06 mm at one day following surgery and it decreased significantly after one week to become 6.47 mm, then it reached the zero level at 14 days and 28 days. This could be explained that in our surgical protocol, a periotome was first utilized to confirm the incision lines in an intention to avoid periosteum shredding that would result from using only the BS1 piezoelectric tip since this tip cuts only hard tissues. Ignoring this particular step before conducting the piezoelectric cuts would be a reason beyond the increased pain levels following surgery found in the previous trials.

Our protocol of pain assessment points was identical to the that of Alikhani et al. who employed a numeric rating scale during piezocision-assisted canine retraction [32]. Although no significant differences were observed between the control group (i.e., no acceleration) and the experimental group in their study, the direct comparison with the current results is not straightforward. This is because of the differences existing in the orthodontic tooth movement strategy, biomechanics and level of force employed; i.e., bilateral canine retraction versus decrowding movements for the six anterior teeth, bodily movement for one tooth in one direction versus three-dimensional multiple derotations, distal tipping, or uprighting for several teeth, and 150–175 g emitted from a NiTi coil spring on each side versus different amounts of force emitted by the aligning NiTi archwires and distributed among six teeth.

A significant difference at one day in the perception of swelling (12.36; *P* = 0.011) was found between the experimental and the control subjects but it decreased significantly after 7 days (*P* = 0.372) reaching the zero level after 14 and 28 days. This could be explained by piezoelectric blade’s precision that permitted fast healing and fine cuttings with minimal morbidity [39]. Even though the difference between the two groups was statistically significant at day one, it cannot be considered clinically important since it did not exceed the assumed threshold of 25-mm difference on the VAS scale.

To the best of our knowledge, no previous trials have evaluated swelling perception following flapless corticotomy. Nevertheless in conventional corticotomy, two previous trials quantified the perception of swelling [18, 27] and revealed higher and longer lasting levels of swelling perception. Al-Naoum et al. mentioned that the proportion of patients who reported medium or severe swelling one day following the corticotomy was 80%, whereas 70% of the patients reported a feeling of mild to moderate swelling after seven days of the operation [18]. Again, the dramatic less percentage of patients suffering from untoward effects can be explained by the minimally invasive nature of flapless piezocision conducted in the current study compared to the traditional technique.

Furthermore, no significant differences were found at one day or at 7 days regarding difficulties of mastication, swallowing or jaws movement limitation. These three possible post-corticotomy functional impairments have not been yet evaluated in the literature employing RCT designs.However, a previous non-controlled cohort prospective study evaluated the oral health-related quality of life using the short-form Oral Health Impact Profile (OHIP-14)-which contained a specific domain focusing on functional impairment [41]. In this study piezoelectric surgery techniques did not significantly modify the OHIP-14 scores at the 3-day (*P* = 0.20) or 7-day (*P* = 0.89) follow-up.

The intervention in the experimental group showed a high level of patients’ acceptance ($\overline{x}$ =86.47 ± 22.47). This could be explained by absence of need for flaps and sutures besides of the less painful and traumatic incisions compared to the conventional methods. This agrees with the results of two previous trials that reported high patients’ acceptability to the piezocision intervention [29, 31].

Recommendation of this procedure to friends was the last question given to the patients in the experimental group. Surprisingly, the answer was positive from all patients. In the same context, the surgical intervention in Mehr’s study did not have any negative effect on patients’ willing to advice friends to undergo a similar procedure [31]. Furthermore, Charavet et al. mentioned that in their experimental group, significantly greater numbers of patients reported that they would undergo the treatment again and that they would recommend it to a friend in comparison with the control group [29].

No significant harms were observed during the entire duration of the study. Patients did not suffer from complications such as lower lip numbness, hematomas, gingival recession or any other short-term post-surgical side effects.

### Limitations

The current trial had several limitations. First, blinding was neither applied to the researcher nor to the patients during this trial, therefore the so-called Hawthorne effect was not filtered out. Actually, it is impossible to employ blinding to patients and health care provides when a surgical intervention is under evaluation. Secondly, there is a need to evaluate periodontal health, teeth vitality and long term post-surgical complications following piezocision surgery. Thirdly, the current two-arm trial compared flapless piezocision-based corticotomy with the conventional orthodontic treatment, but the missing link was a separate group of patients undergoing conventional corticotomy. If such a three-arm design was accomplished, we would have been in a higher position to compare patient-centered outcomes between the three groups with different levels of invasiveness. Finally, the assessment of pain and discomfort with other acceleration methods (e.g. physical, surgical, mechanical) in conjunction with different orthodontic tooth movement strategies such as upper incisors retraction, canine retraction, and en masse retraction should be also covered in future research work.

## Conclusions

No significant differences in the levels of pain and discomfort were found between the piezocision-assisted flapless corticotomy group and the conventional orthodontic treatment group. However, a slight significant difference was found regarding patients’ perception of swelling at one day following treatment commencement and this difference decreased significantly within seven days. The patient-centered outcomes revealed a high level of acceptance and satisfaction with this technique.

## Additional files

Additional file 1: Pain and discomfort questionnaire. (DOCX 154 kb)

Additional file 2: Satisfaction questionnaire. (DOCX 155 kb)

## Abbreviations

ExpG: Experimental group
RCT: Randomized controlled trial
VAS: Visual analog scale
