# Supplementary material for: Evaluation of the levels of pain and discomfort of piezocision-assisted flapless corticotomy when treating severely crowded lower anterior teeth: a single-center, randomized controlled clinical trial
Source: BMC Oral Health. 2019 Apr 16;19:57. doi: 10.1186/s12903-019-0758-9 (PMC6469154; doi:10.1186/s12903-019-0758-9)

**Pain and Discomfort Questionnaire**

- How much pain did you have?

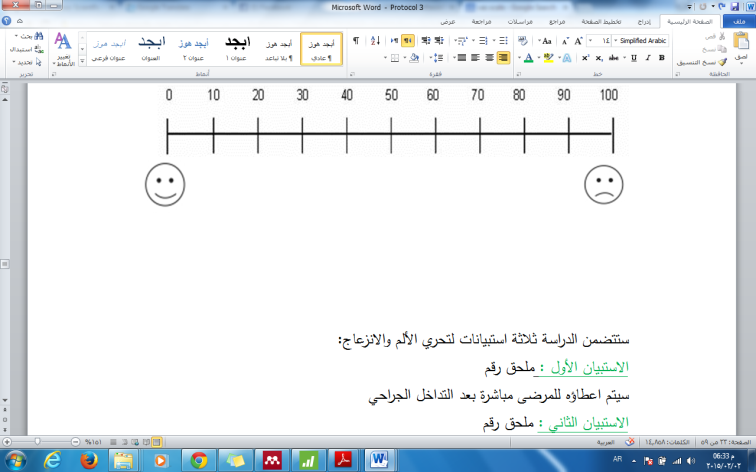

- How much discomfort did you have?

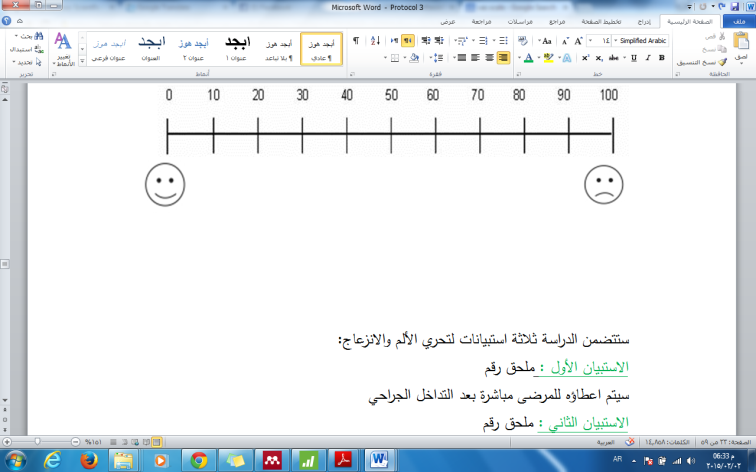

- How much swelling did you feel?

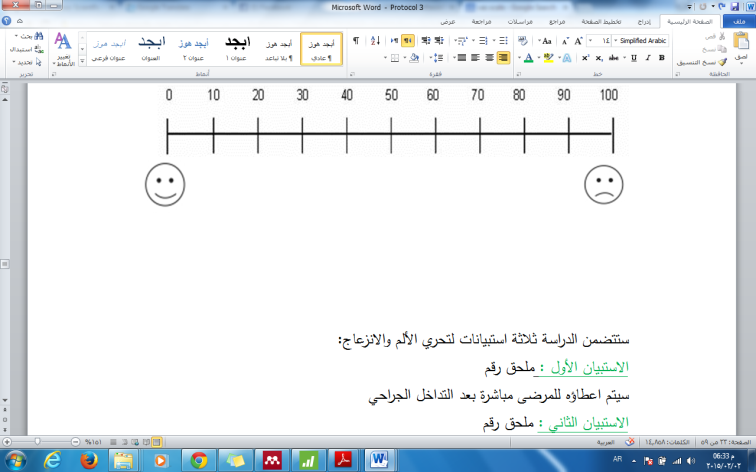

- How much difficulties in mastication did you have?

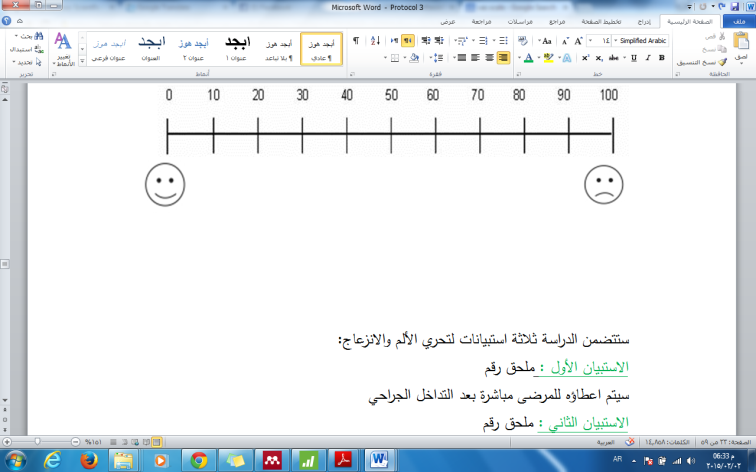

- How much difficulties in swallowing did you feel?

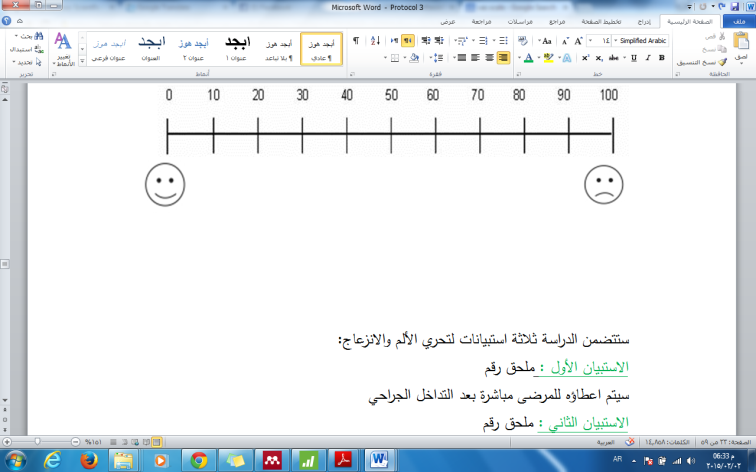

- How much mouth limitation did you feel?

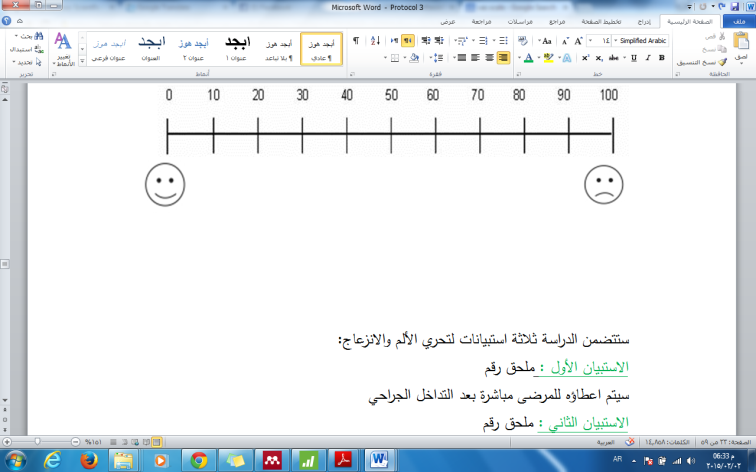

Supplement: Supplementary file 1 — Pain and discomfort questionnaire. (DOCX 154 kb) [file 12903_2019_758_MOESM1_ESM.docx]
