# Supplementary material for: Evaluation of the levels of pain and discomfort of piezocision-assisted flapless corticotomy when treating severely crowded lower anterior teeth: a single-center, randomized controlled clinical trial
Source: BMC Oral Health. 2019 Apr 16;19:57. doi: 10.1186/s12903-019-0758-9 (PMC6469154; doi:10.1186/s12903-019-0758-9)
Supplement: Supplementary file 2 — Satisfaction questionnaire. (DOCX 155 kb) [file 12903_2019_758_MOESM2_ESM.docx]

**Satisfaction questionnaire**

- How much are you satisfied with your accelerated treatment?

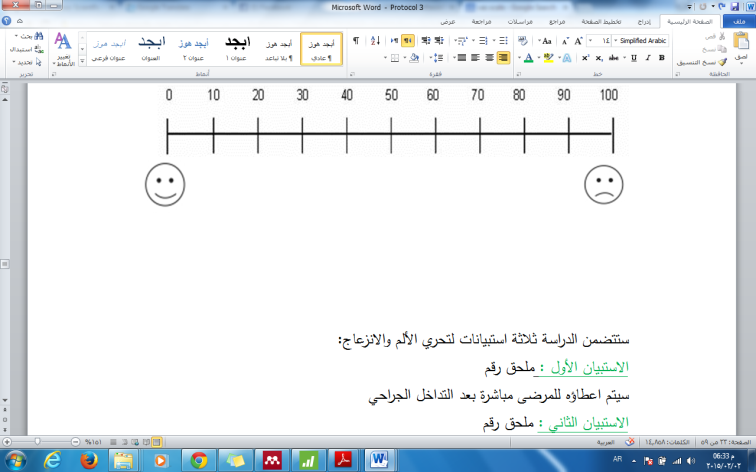


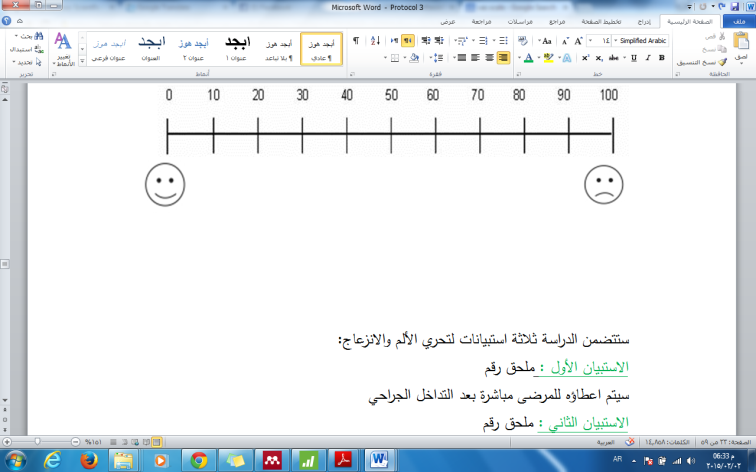

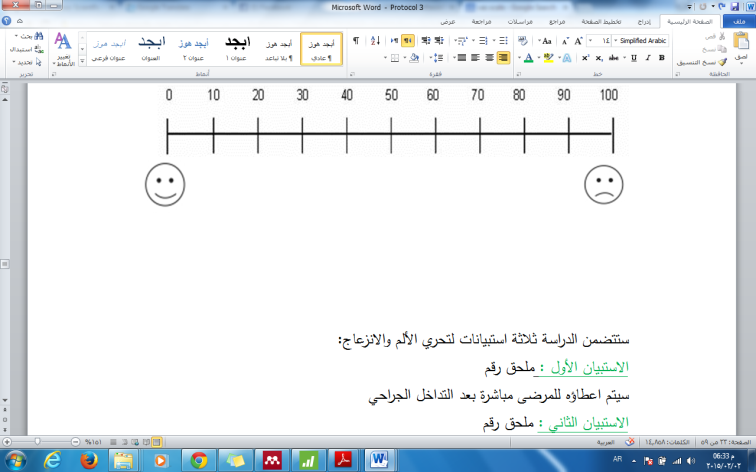


- Would you recommend this procedure to a friend?

1-YES 2-NO

- Did you take any type of pain killers during the treatment?
  1-YES 2-NO

If the answer is yes, when did you take pain killers? How many tablets as a whole?
